# Claw hands in acute intermittent porphyria

**DOI:** 10.1093/omcr/omaf266

**Published:** 2025-12-26

**Authors:** Jiaan-Der Wang, Cheng-Ta Chou

**Affiliations:** Center for Rare Disease and Hemophilia, Children’s Medical Center, Taichung Veterans General Hospital, 1650 Taiwan Boulevard Sect. 4, Taichung City 407219, Taiwan; Department of Post-Baccalaureate Medicine, College of Medicine, National Chung Hsing University, 145 Xingda Rd., South Dist., Taichung City 402202, Taiwan; Neurological Institute, Taichung Veterans General Hospital, 1650 Taiwan Boulevard Sect. 4, Taichung City, 407219, Taiwan

A 48-year-old female with acute intermittent porphyria (AIP)—a rare multisystemic disorder characterized by recurrent acute attacks and chronic complications [1] —presented with severe ‘claw hands’ due to chronic porphyric neuropathy.

She experienced recurrent cramp-like abdominal pain and progressive upper limb weakness, a hallmark of porphyric neuropathy. The weakness progressed from distal to proximal and was more pronounced on the right side. Chronic neurological symptoms included intermittent involuntary movements, paresthesia, impaired speech, and paralysis of all limbs.

A nerve conduction velocity test revealed severe motor and sensory axonal polyneuropathy. Her hands exhibited hyperextension at the metacarpophalangeal (MCP) joints and flexion at the proximal interphalangeal (PIP) and distal interphalangeal (DIP) joints of the 4^th^ and 5^th^ fingers—classic ‘claw hands’—with atrophy of the hypothenar eminence and interossei muscles (Fig. 1A). Muscle strength was impaired in adduction, abduction, and flexion of the DIP joints of the 4^th^ and 5^th^ fingers.

**Figure 1:**
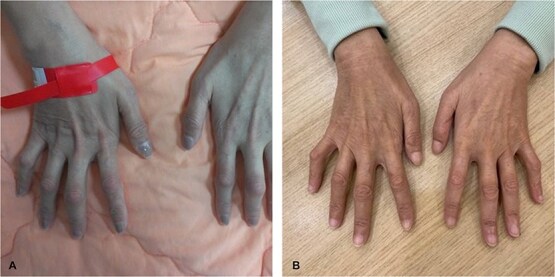
(A) A 48-year-old female with AIP exhibited classic ‘claw hands’ deformity characterized by hyperextension at the metacarpophalangeal (MCP) joints and flexion at the proximal (PIP) and distal interphalangeal (DIP) joints of the 4th and 5th fingers. (B) under givosiran treatment, flexion of the PIP and DIP joints of the 4th and 5th fingers improved. The atrophy of the hypothenar eminence and interossei muscles also improved.

Diagnosed with AIP in 2011, she started prophylactic treatment with human hemin and glucose in 2014. In 2018, she was enrolled in a Phase 3 clinical trial of givosiran, an RNAi therapeutic targeting aminolevulinic acid synthase 1 (ALAS1) [2].

Prior to givosiran, her composite annualized attack rate (AAR) was 24. Under treatment, the AAR dropped to zero. Accordingly, flexion of the PIP and DIP joints of the 4th and 5th fingers improved, as did the atrophy of the hypothenar eminence and interossei muscles. (Fig. 1B).

This case highlights the importance of managing both acute attacks and chronic complications. Givosiran reduces aminolevulinic acid (ALA) and porphobilinogen (PBG) levels, thereby mitigating neurotoxic effects that contribute to porphyric neuropathy. This case underscores the need to look beyond focal neuropathies, as claw hands may mimic ulnar palsy but reflect an underlying systemic disorder like acute intermittent porphyria.

## Funding statement

This publication was under courtesy review and the publication fee was funded by Alnylam Pharmaceuticals.

## Consent

Written informed consent for publication of their clinical details and/or clinical images was obtained from the patient.

## Guarantor

Jiaan-Der Wang.
